# Applications and Performance of Artificial Intelligence in Spinal Metastasis Imaging: A Systematic Review

**DOI:** 10.3390/jcm14165877

**Published:** 2025-08-20

**Authors:** Vivek Sanker, Poorvikha Gowda, Alexander Thaller, Zhikai Li, Philip Heesen, Zekai Qiang, Srinath Hariharan, Emil O. R. Nordin, Maria Jose Cavagnaro, John Ratliff, Atman Desai

**Affiliations:** 1 Department of Neurosurgery, Stanford University, Palo Alto, CA 94305, USA; hsrinath@stanford.edu (S.H.); enordin@stanford.edu (E.O.R.N.); mjcava@stanford.edu (M.J.C.); jratliff@stanford.edu (J.R.); atman@stanford.edu (A.D.); 2 Department of Neurosurgery, St John’s Medical College, Bangalore 560034, Karnataka, India; poorvikha2712@gmail.com; 3 Department of Neurosurgery, Medical University of Graz, 8010 Graz, Styria, Austria; alexander.thaller@medunigraz.at; 4 Department of Clinical Neurosciences, Addenbrooke’s Hospital, Cambridge CB2 0QQ, UK; zl498@cam.ac.uk; 5 Faculty of Medicine, University of Zurich, 8032 Zurich, Switzerland; heesenphilip99@gmail.com; 6 School of Medicine and Population Health, University of Sheffield Medical School, Sheffield S10 2RX, UK

**Keywords:** artificial intelligence (AI), spinal metastasis, diagnostic imaging, deep learning, convolutional neural networks (CNNs), radiomics, machine learning

## Abstract

**Background:** Spinal metastasis is the third most common site for metastatic localization, following the lung and liver. Manual detection through imaging modalities such as CT, MRI, PET, and bone scintigraphy can be costly and inefficient. Preliminary artificial intelligence (AI) techniques and computer-aided detection (CAD) systems have attempted to improve lesion detection, segmentation, and treatment response in oncological imaging. The objective of this review is to evaluate the current applications of AI across multimodal imaging techniques in the diagnosis of spinal metastasis. **Methods:** Databases like PubMed, Scopus, Web of Science Advance, Cochrane, and Embase (Ovid) were searched using specific keywords like ‘spine metastases’, ‘artificial intelligence’, ‘machine learning’, ‘deep learning’, and ‘diagnosis’. The screening of studies adhered to the PRISMA guidelines. Relevant variables were extracted from each of the included articles such as the primary tumor type, cohort size, and prediction model performance metrics: area under the receiver operating curve (AUC), accuracy, sensitivity, specificity, internal validation and external validation. A random-effects meta-analysis model was used to account for variability between the studies. Quality assessment was performed using the PROBAST tool. **Results:** This review included 39 studies published between 2007 and 2024, encompassing a total of 6267 patients. The three most common primary tumors were lung cancer (56.4%), breast cancer (51.3%), and prostate cancer (41.0%). Four studies reported AUC values for model training, 16 for internal validation, and five for external validation. The weighted average AUCs were 0.971 (training), 0.947 (internal validation), and 0.819 (external validation). The risk of bias was the highest in the analysis domain, with 22 studies (56%) rated high risk, primarily due to inadequate external validation and overfitting. **Conclusions:** AI-based approaches show promise for enhancing the detection, segmentation, and characterization of spinal metastatic lesions across multiple imaging modalities. Future research should focus on developing more generalizable models through larger and more diverse training datasets, integrating clinical and imaging data, and conducting prospective validation studies to demonstrate meaningful clinical impact.

## 1. Introduction

Spinal metastasis represents the third most common site for metastatic localization, following the lung and the liver [1]. Nearly 70% of the primary breast and prostate tumors are metastasized to the axial skeleton due to its high red marrow content which can in turn lead to a spectrum of clinical manifestations and immensely impact a patient’s quality of life. Complications include pathological fractures, spinal cord compression, spinal deformity, and reduced mobility and neurological deficits, which can progress and be irreversible without early treatment [2]. Therefore, early detection and diagnosis play a pivotal role in clinical practice [3]. Advanced imaging techniques such as computed tomography (CT), magnetic resonance imaging (MRI), positron emission tomography (PET), and bone scintigraphy are typically used in assessing osseous metastases. The sensitivity and specificity of CT, MRI, PET and bone scintigraphy are reported to be 79.2% and 92.3%, 94.1% and 94.2%, 89.8% and 63.3%, and 80.0% and 92.8%, respectively [4].

The detection of spinal metastasis through these various imaging modalities is often time-consuming and challenging. Recently, preliminary artificial intelligence (AI) techniques and computer-aided detection (CAD) software systems have been applied in medical imaging applications, particularly in oncological imaging [5]. The use of machine learning techniques such as radiomics-based feature analysis and convolutional neural networks (CNNs) have been studied for lesion detection, segmentation, and treatment response [6]. Radiomics, in principle, is capable of extracting subtle features from images beyond those inferable to the human eye, but one disadvantage is that it requires the extraction of handcrafted features [7]; however, this can potentially be circumvented by employing deep learning techniques which learn important imaging features for classification through a hierarchical architecture, as demonstrated by CNN. Thus, automated lesion detection could potentially improve sensitivity for detecting bone metastases [8,9].

However, the field of AI has progressed rapidly in recent years, and the application of AI in spinal metastasis detection remains nascent. The objective of this article is to review the various AI models utilizing multimodal imaging techniques to detect spinal metastasis.

## 2. Materials and Methods

### 2.1. Ethical Review

Ethical review and approval were waived for this study due to it being a systematic review of previously published data and did not involve human participants or the collection of new data.

### 2.2. Search Strategy

We searched PubMed, Scopus, Web of Science Advance, Cochrane, and Embase (Ovid) databases to identify relevant studies, using a search query with specific keywords like ‘spine metastases’, ‘artificial intelligence’, ‘machine learning’, ‘deep learning’, and ‘diagnosis’ (Supplementary Table S1). The population under consideration included adults. The objective was to identify studies reporting the use of AI/DL models in predicting diagnosis and image analysis in spine metastases.

Irrelevant articles, such as studies unrelated to spinal metastases and those purely investigating primary spinal tumors, were excluded. Animal studies, reviews, and non-original research articles were also excluded from our analysis to ensure the inclusion of primary research data relevant to our objective. The electronic search ranged from the period’s earliest available date up to 27 January 2025 [8].

### 2.3. Screening of Studies

**Figure 1:**
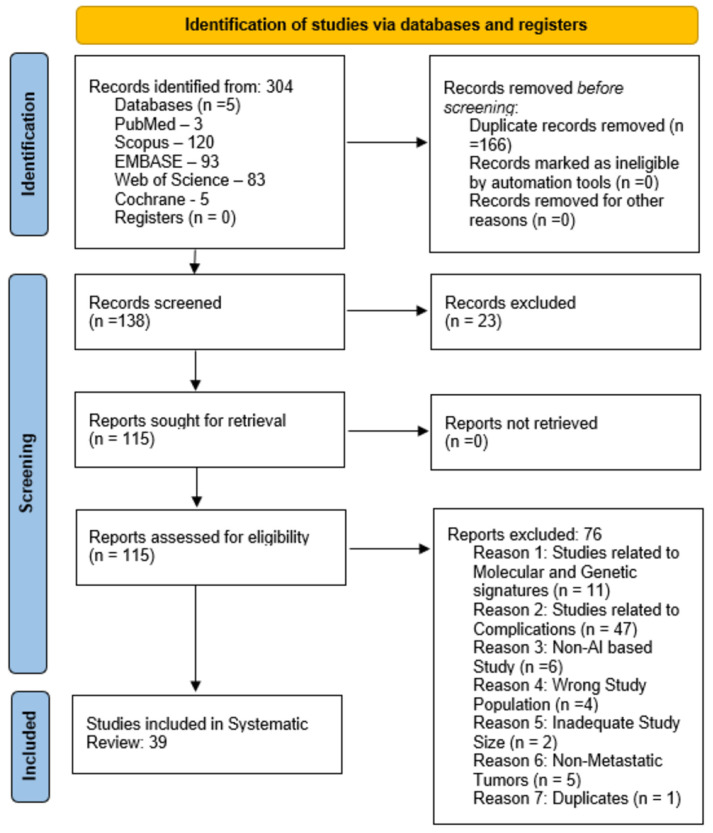
PRISMA flow diagram. A PRISMA flow diagram is presented to illustrate the screening of studies leading to 39 studies being included in the systematic review.

Each study’s title and abstract were screened for relevance before proceeding to full-text screening, which was independently assessed by two reviewers (P.G. and V.S.). Any discrepancies were addressed through consultation with a third reviewer (S.H.). The screening of studies adhered to the PRISMA (Preferred Reporting Items for Systematic Reviews and Meta-Analyses) guidelines (Figure 1).

### 2.4. Data Extraction

Three independent authors (P.G., V.S., and A.T.) extracted relevant data from the included studies. The data collected included study design, participant demographics, and the number of participants with respective outcomes and complications. Discrepancies in data extraction were resolved through consensus [8].

### 2.5. Data Analysis

Relevant variables were extracted from each of the included articles such as the primary tumor type, cohort size, prediction model performance matrices—area under the receiver operating characteristic curve (AUC)—and type of validation (internal or external validation). The weighted average of the AUC was calculated. All statistical analyses were conducted using Excel, R Statistical Software, version 4.3.1, and Python, version 3.13.3.

### 2.6. Quality Assessment

The quality assessment was performed using the PROBAST (Prediction model Risk of Bias Assessment Tool (Figure 3 and Figure 4).

PROBAST is designed for assessing the risk of bias and applicability in studies that develop, validate, or update predictive models. It is a structured tool that assesses four domains:

Participants: Evaluating whether the data sources or patient samples used for training and testing are appropriate and representative of the clinical population. Predictors: Ensuring that input data or predictors are well defined and appropriately measured. Outcome: Ensuring that the outcomes (e.g., model predictions, decisions) are clearly defined and relevant to clinical scenarios. Analysis: Evaluating whether the model performance metrics, training/validation processes, and statistical analysis methods are robust and unbiased.

## 3. Results

This review encompasses 39 studies published between 2007 and 2024, including a total of 6267 patients with a median of 88 patients per study, ranging from 3 to 941 patients. Model performance metrics included AUC, accuracy, sensitivity, specificity, internal validation, and external validation. AUC was most commonly reported, which is why we focused our analysis on that. Out of the 39 studies, 9 reported the number of lesions observed in their study cohorts, with a median of 137 lesions per study. Out of the 39 studies, 12 reported the number of scans performed, with a median of 405.5 scans per study (Table 1).

**Figure 2:**
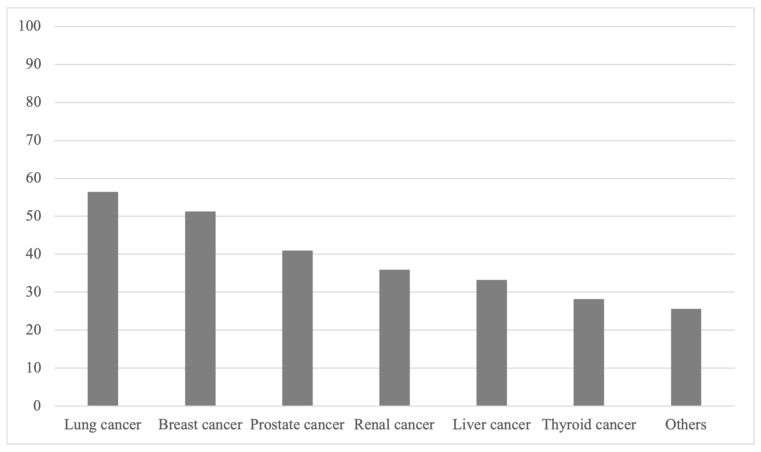
Percentage of studies featuring primary tumor types. The percentage of how many studies feature specific primary tumor types is presented. A wide variety of different cancer types were analyzed in the studies included with lung cancer and breast cancer being represented most often.

Among the 39 studies, the three most common primary tumor types were lung cancer, breast cancer, and prostate cancer, as reported in 22 (56.4%), 20 (51.3%), and 16 (41.0%) studies, respectively (Table 2 and Figure 2). In contrast, neuroendocrine tumors, bladder cancer, and sarcoma were the least common, each reported in two (5.1%), three (7.7%), and three (7.7%) studies, respectively (Table 2). In addition, we also analyzed the primary data modalities used to train and validate the AI models across all 39 studies. Out of the 39 studies included in our systematic review, 20 studies (51.3%) used magnetic resonance imaging (MRI) as the primary data source for their models. In comparison, 17 studies (43.6%) utilized computed tomography (CT), whereas the two remaining studies were based on non-imaging data, such as clinical text for a large language model (LLM) or clinical variables for survival prediction [10,11].

Among the 39 studies, 4 reported AUC values for the established models from the training of the model (Table 3), 16 reported AUC values for the established models from an internal validation (Table 4) and five studies reported AUC values from an external validation (Table 5).

The weighted average AUC value among the four studies that reported AUC values and the corresponding 95% confidence intervals for the training of the established models is 0.971 (95% CI: 0.965–0.978). Wherever 95% confidence intervals were not reported, the weighted average of the reported 95% confidence intervals was used.

The weighted average AUC value among the 16 studies that reported AUC values and the corresponding 95% confidence intervals for the internal validation of the established models is 0.947 (95% CI: 0.935–0.958). Wherever 95% confidence intervals were not reported, the weighted average of the reported 95% confidence intervals was used.

The weighted average AUC value among the five studies that reported AUC values and the corresponding 95% confidence intervals for the external validation of the established models is 0.819 (95% CI: 0.797–0.840). Wherever the 95% confidence intervals were not reported, the weighted average of the reported 95% confidence intervals was used.

### 3.1. Risk of Bias Assessment

**Figure 3:**
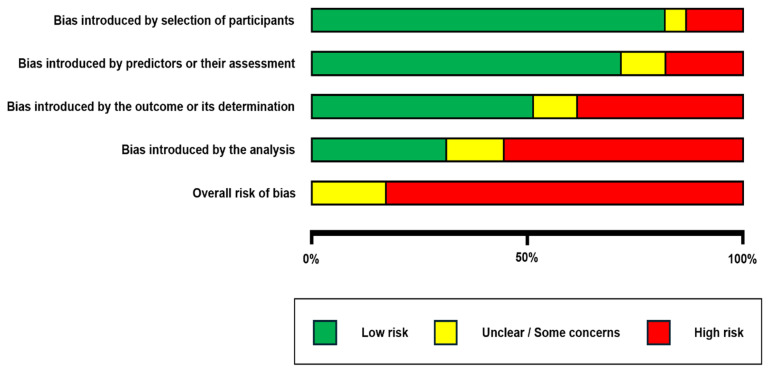
A risk of bias analysis is presented.

**Figure 4:**
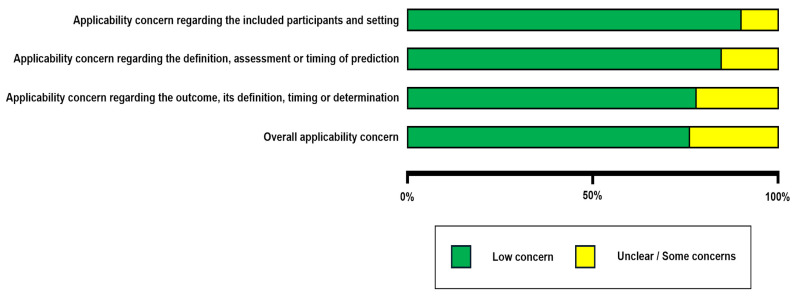
An applicability analysis of the studies included is presented.

The risk of bias (Figure 3) and applicability (Figure 4) concerns of the 39 included studies were evaluated using the PROBAST tool, focusing on AI-based predictive models for spinal metastasis diagnosis and image analysis. In the participants domain, 32 studies (82%) demonstrated low risk of bias, with appropriate patient selection and representative cohorts, while five (13%) had high risk and two (5%) had unclear risk, primarily due to the small sample sizes or non-representative populations. For the predictors domain, 28 studies (72%) exhibited low risk with well-defined imaging features, whereas seven (18%) had high risk and four (10%) had unclear risk, often linked to inconsistent feature extraction or unclear predictor definitions. The outcome domain revealed greater variability, with 20 studies (51%) at low risk, 15 (38%) at high risk, and four (10%) at unclear risk; high-risk studies frequently lacked standardized reference standards for metastasis diagnosis or suffered from subjective outcome assessment. The analysis domain presented the most significant concerns, with only 12 studies (31%) at low risk, 22 (56%) at high risk, and five (13%) at unclear risk. Common issues included inadequate external validation, overfitting, and the poor handling of missing data, undermining model reliability and generalizability.

Regarding applicability, 35 studies (90%) had low concerns in the participants domain, 33 (85%) in the predictors domain, and 30 (77%) in the outcome domain, indicating relevance to the review’s focus on spinal metastasis imaging. However, high applicability concerns in a minority of studies arose from niche cohorts (e.g., specific tumor subtypes) or experimental imaging modalities not widely translatable to clinical practice. Overall, 15 studies (38%) were deemed to have high risk of bias in at least one domain, predominantly the analysis domain, highlighting critical limitations in the current evidence base that may overestimate model performance and warrant the cautious interpretation of reported outcomes.

### 3.2. Temporal Overview of Included Studies

The 39 included studies were stratified into two temporal groups: those published before 2020 (n = 9) and those published from 2020 onward (n = 30). This analysis revealed a substantial acceleration in research output, with 77% of the studies in this review being published since the beginning of 2020, reflecting rapid advancements in deep learning and computational resources in recent years.

## 4. Discussion

This systematic review examined 39 studies published between 2007 and 2024, providing a comprehensive evaluation of artificial intelligence applications in spinal metastasis imaging. The findings demonstrate relatively high-performance metrics across various AI methodologies, with weighted average AUC values of 0.971, 0.947, and 0.819 for training, internal validation, and external validation, respectively. These results overall suggest diagnostic potential while highlighting the expected performance drop during validation phases that warrant further consideration. Furthermore, our analysis reveals that deep learning approaches have become increasingly prevalent, with convolutional neural networks (CNNs) and their architectural variants representing the dominant methodologies. This trend aligns with broader developments in medical imaging, where deep learning has demonstrated particular efficacy in semantic segmentation tasks—delineating both vertebral structures and metastatic lesions with precision approaching expert radiologists [28].

Our findings are consistent with a recent meta-analysis, like Papalia et al. [29] and Tao et al. (2025) [30], with the latter reporting pooled sensitivity and specificity values of 0.88 [0.82–0.92] and 0.89 [0.84–0.93], respectively, across AI models for bone metastasis detection [9]. Notably, in this study, deep learning approaches demonstrated a marginally superior performance compared to traditional machine learning methods, with a pooled AUC of 0.95 versus 0.93. This slight performance advantage likely reflects deep learning’s capacity for automated feature extraction, potentially capturing subtle imaging biomarkers that may elude conventional radiomic approaches. The relatively consistent performance across different AI architectures suggests that metastatic lesions exhibit reasonably distinctive imaging features that various algorithms can effectively identify. However, we observed variability in model performance between training and validation cohorts (AUC drop from 0.971 to approximately 0.947 (internal validation) and 0.819 (external validation)), which highlights ongoing challenges in developing generalizable models capable of maintaining performance across heterogeneous patient populations and imaging protocols.

The observed performance drop, particularly during external validation (AUC 0.819 versus 0.971 in training) closely mirrors the analytic deficiencies identified by our PROBAST assessment. The high risk of bias in the ‘analysis’ domain—most notably overfitting, optimistic performance estimates due to inappropriate internal validation techniques, and absent external validation—directly contributes to this degradation. These methodological shortcomings artificially inflate performance during model development, but fail to ensure robustness when tested on new datasets, thus undermining clinical reliability. The discrepancy in validation outcomes therefore reflects not only natural variability, but preventable design limitations. This underscores the importance of rigorous validation practices, including representative external cohorts and transparent reporting, as prerequisites for generalizable AI in spinal metastasis imaging.

Spinal metastasis imaging encompasses multiple modalities, each with distinct advantages. While MRI remains the gold standard with reported sensitivity and specificity exceeding 98% for metastatic lesion detection [31], AI applications span across CT, MRI, PET-CT, and hybrid approaches. Our findings suggest that AI models demonstrate promising performance across these modalities, though direct cross-modality comparisons remain limited by methodological heterogeneity. Another interesting finding in our study is the near-even split in the use of MRI and CT as the primary imaging modalities for AI model development. The slight predominance of MRI-based studies over the widely available CT modality may reflect its superior soft-tissue contrast, critical for assessing tumor characteristics, thereby providing a comprehensive diagnostic picture for clinicians.

Performance variability across different types of lesions merits particular attention. The literature suggests differential performance between osteolytic and osteoblastic metastases, likely reflecting their distinct radiographic presentations [9,23]. Osteolytic lesions typically present with greater contrast against surrounding bone, potentially facilitating more reliable detection compared to the more subtle density changes characteristic of sclerotic metastases [32]. This differential performance has implications for primary tumor-specific applications, given the propensity of certain malignancies [e.g., prostate cancer] toward osteoblastic metastases versus the predominantly osteolytic pattern seen in others [e.g., lung cancer]. Therefore, while the overall diagnostic accuracy reported in reviews like Tao et al. (2025) [30] is high, our findings emphasize that this performance may not be uniform across all metastatic subtypes [30].

Automated systems for lesion detection and characterization may potentially enhance radiologist and clinician performance, particularly for less experienced practitioners by providing a second opinion. Observer studies have demonstrated that AI assistance can significantly improve detection rates, with Ong et al. (2022) reporting improved figure of merit scores for both attending physicians [0.848 to 0.876, *p* = 0.01] and residents [0.752 to 0.799, *p* = 0.02] when assisted by AI algorithms [9]. Another promising application involves reducing false positive rates in metastasis detection. Wang et al. demonstrated a 44.8% reduction in false positives using a Siamese neural network architecture, potentially alleviating a significant challenge in conventional imaging interpretation [33]. This improvement could substantially impact clinical workflow by reducing unnecessary follow-up imaging and interventions prompted by false positive findings. Beyond detection, segmentation represents another valuable clinical application. The accurate delineation of metastatic lesions is crucial for treatment planning, particularly for targeted radiation therapy and minimally invasive interventions. The high dice similarity coefficients reported by Arends et al. [97% and 95% for internal and external validation] show that automated segmentation approaches can produce an impressive performance that warrants direct comparison studies to expert manual segmentation [34]. Such capabilities could significantly improve workflow efficiency while maintaining high accuracy in treatment planning, facilitating the multidisciplinary management of spinal tumors [35]. This shift towards clinically relevant models is a very recent trend, as our temporal analysis confirms: a majority of the included papers were published after 2020. The foundational studies published before 2020 often relied on classical machine learning or computer-aided detection (CAD) systems, which, although pioneering, reflect the technological landscape of an earlier time. In contrast, studies published from 2020 onward predominantly leverage more sophisticated deep learning architectures, which generally report higher diagnostic accuracy and more robust segmentation capabilities. As the field of AI matures rapidly from exploratory concepts to more clinically translatable, high-performance models, these studies are more representative of the current capabilities of AI.

Despite promising results, several challenges currently limit the clinical translation of AI for spinal metastasis imaging. Foremost among these is the persistent gap between training and validation performance metrics, suggesting potential overfitting or limited generalizability. The weighted average AUC decreased from 0.971 during training to 0.947 during internal validation and 0.819 during external validation, highlighting the need for more robust validation approaches and diverse training datasets. Additionally, the pooled AUCs presented in Table 3, Table 4 and Table 5 need to be interpreted cautiously as they reflect the weighted averages of AUCs of highly varied prediction tasks and thus provide a general overview of AI’s current capability of diagnosing spinal metastases, yet they do not allow for significant statements about specific prediction tasks to be made. Furthermore, given the nature of the missing statistical data, wherever 95% confidence intervals were not reported, the weighted average of the reported 95% confidence intervals was used to calculate the weighted average AUC with its corresponding 95% confidence interval. Such imputation may, however, underestimate the true uncertainty of the studies with unreported 95% confidence intervals. This could have led to bias in study weighting, potentially artificially narrowing pooled confidence intervals, especially if the unreported data were from smaller or lower-quality studies.

Data limitations represent another significant challenge. The median cohort size of 88 patients across the reviewed studies indicates relatively modest training datasets by contemporary deep learning standards. This limitation is particularly relevant given the heterogeneity of spinal metastatic disease across the different primary malignancies, imaging protocols, and patient demographics. The predominance of certain primary malignancies in the literature [lung 56.4%, breast 51.3%, prostate 41.0%] may also limit generalizability to less common primary tumors. AI models perform differentially across lesion types. Given that certain kinds of primary tumors tend to give rise to either osteolytic or osteoblastic metastases, the majority of models may have been optimized to detect lesions more commonly found with their respective primary tumor types. Osteolytic metastases (common in lung/breast cancer) show higher detection rates than osteoblastic lesions (typical in prostate cancer), reflecting radiographic contrast differences [36]. This impacts a mixed-dataset training where prostate cancer constituted 41% of studies. This may lead to reduced accuracy in the detection of metastatic lesions with atypical radiographic presentation. Technical challenges in metastasis detection warrant consideration as well. Differentiating metastatic lesions from benign processes such as degenerative changes, hemangiomas, and other non-malignant entities remains challenging even for expert radiologists. While AI may excel at pattern recognition, distinguishing subtle differences between malignant and benign lesions with similar imaging characteristics represents an ongoing challenge.

Several promising directions emerge for future research in this field. First, multimodal approaches integrating complementary information from different imaging sequences [e.g., T1, T2, STIR, diffusion-weighted imaging in MRI] may improve detection sensitivity and specificity. Wallace et al. (2015) highlighted the superiority of multisequence MRI in detecting marrow infiltration before overt trabecular or cortical destruction becomes apparent, suggesting the potential benefits from AI models capable of integrating information across multiple sequences [37]. Second, the integration of clinical data with imaging findings may enhance the overall diagnostic performance. Current AI approaches predominantly focus on image analysis in isolation, neglecting potentially valuable clinical information such as primary tumor characteristics, treatment history, and laboratory parameters. Multimodal models incorporating both imaging and clinical data may better reflect the integrative approach used by clinicians in practice [38]. Third, federated learning approaches represent a promising strategy to address data limitations while preserving patient privacy. Data augmentation techniques have proven effective in bone imaging applications. Various augmentation strategies including geometric transformations (rotation, scaling, flipping), intensity modifications, and elastic deformations have been reported in literature. Advanced augmentation techniques such as generative adversarial networks (GANs) and synthesis-based augmentation have shown promise for creating realistic bone lesion variations while preserving clinical relevance [39]. Multiple studies have successfully employed transfer learning approaches, particularly domain-specific transfer learning within medical imaging. Transfer learning from medical imaging datasets (rather than natural image datasets like ImageNet) can produce significant performance improvements with small training datasets. For bone metastasis detection, transfer learning from related skeletal imaging tasks has shown superior results compared to ImageNet-based initialization [39]. By enabling model training across multiple institutions without centralized data sharing, such approaches could significantly expand the available training data while addressing the privacy concerns inherent in cross-institutional collaboration. Finally, prospective clinical validation studies are essential to demonstrate meaningful clinical impact beyond retrospective performance metrics. Such studies should not only evaluate diagnostic accuracy but also effects on clinical decision making, workflow efficiency, and ultimately patient outcomes.

This systematic review has several strengths, including comprehensive database searches, rigorous methodology following PRISMA guidelines, and a detailed extraction of performance metrics across a diverse range of AI applications. The inclusion of studies spanning nearly two decades provides a robust overview of the evolving landscape of AI in spinal metastasis imaging. However, several limitations warrant consideration. First, the heterogeneity between the included studies complicates direct comparison. The use of a wide range of AI architectures including traditional machine learning, convolutional neural networks, and transformer-based models, imaging modalities such as CT, MRI, and PET, as well as varying validation techniques including split-sample validation and k-fold cross-validation may affect the pooled results. This is because performance metrics are dependent on the underlying model architecture, the nature and quality of the data included, and the rigor of the validation method used. Significant heterogeneity therefore limits the conclusions that can be drawn from the pooled AUCs about specific prediction tasks. This especially holds true when factoring in the second limitation in the form of publication bias, which may have influenced the reported results, with negative or inconclusive studies potentially less likely to be published. Third, the rapid evolution of AI methodologies means that some of the earlier included studies may utilize techniques that are now considered suboptimal compared to state-of-the-art approaches.

## 5. Conclusions

AI-based approaches show substantial promise for enhancing the detection, segmentation, and characterization of spinal metastatic lesions across multiple imaging modalities. While technical challenges and implementation barriers remain, continued methodological refinements and rigorous clinical validation may soon translate these promising research findings into valuable clinical tools. Future research should focus on developing more generalizable models through larger and more diverse training datasets, integrating clinical and imaging data, multi-institutional data sharing and federated learning, and conducting prospective validation studies to demonstrate meaningful clinical impact.

## Figures and Tables

**Table 1:** An overview of the studies analyzed is presented, showcasing a set of heterogeneous studies published between 2007 and 2024.

Year of publishing (range)	2007–2024
Total number of patients	6267
Median number of patients	88
Number of patients per study (range)	3–941
Median number of lesions	137
Median number of scans	405.5

**Table 2:** The frequency of primary tumor types is shown. Lung cancer proved to be the most common type of cancer analyzed, whereas neuroendocrine tumors were the least common.

Primary Tumor Type	Number of Studies (Percentage)
Lung cancer	22/39 (56.4%)
Breast cancer	20/39 (51.3%)
Prostate cancer	16/39 (41.0%)
Bladder cancer	3/39 (7.7%)
Sarcoma	3/39 (7.7%)
Neuroendocrine tumors	2/39 (5.1%)

**Table 3:** The AUC (training of each study’s best model) and corresponding 95% confidence interval of studies reporting AUC values for the established radiomics models for the training of the model is presented. Three of four studies yielded an AUC of >0.93. ^1^ = Magnetic resonance imaging.

Study	Output/Prediction	Best Performing Model	AUC	95% CI
Wang et al. (2024) [12]	Prediction of vertebral volumetric bone mineral density in spinal metastases	Deep learning (3DResUNet)	0.977	0.970–0.984
Duan et al. (2023) [13]	Identification of origin for spinal metastases from MRI ^1^	Deep learning	0.94	-
Zhang et al. (2023) [14]	Differentiating spinal metastases from multiple myeloma	Radiomics nomogram	0.856	0.804–0.907
Liu et al. (2022) [15]	Differentiating spinal metastases from multiple myeloma	Radiomics (2EPV-CFS-model)	0.94	-

**Table 4:** The AUC (internal validation of each study’s best model) and corresponding 95% confidence interval of studies reporting AUC values for the established radiomics models for the internal validation of the model is presented. AUCs range from 0.76 to 1.00. ^1^ = split sample internal validation, ^2^ = k-fold cross validation, ^3^ = Computer tomography, ^4^ = Bagging combined with a REPTree.

Study	Output/Prediction	Best Performing Model	AUC		95% CI	
Ahn et al. (2024) [16]	Uncertainty quantification in automated detection of vertebral metastasis	Ensemble Monte Carlo dropout (EMCD; deep learning model)	0.93 ^1^		-	
Wang et al. (2024) [12]	Prediction of vertebral volumetric bone mineral density in spinal metastases	Deep learning (3DResUNet)	0.966 ^1^		0.944–0.988 ^1^	
Duan et al. (2023) [13]	Identification of origin for spinal metastases from MRI ^1^	Deep learning	0.76 ^2^		-	
Duan et al. (2023) [17]	Differentiating spinal tuberculosis and spinal metastases	Multiscale vision transformers V2 (MVITV2)	0.98 ^2^		-	
Koike et al. (2023) [18]	Detection and classification of lytic-dominant lesions	Deep learning-based computer-aided detection system	0.941 ^1^		-	
Li et al. (2023) [19]	Differentiating solitary metastasis and solitary primary tumor	Radiomics nomogram	0.980 ^2^	0.924 ^1^	0.959–0.995 ^2^	0.693–0.916 ^1^
Liu et al. (2023) [20]	Prediction of primary tumor sites in spinal metastases	ResNet-50 CNN (deep learning)	0.77 ^2^		-	
Shi et al. (2023) [21]	Differentiating spinal osteolytic metastases from multiple myeloma	XGBoost with multiparameter DECT (mpDECT)	1.00 ^2^	0.97 ^1^	-	-
Zhang et al. (2023) [14]	Differentiating spinal metastases from multiple myeloma	Radiomics nomogram	0.853 ^1^		0.764–0.919 ^1^	
Chen et al. (2022) [22]	Differentiating spinal metastases from multiple myeloma	Multi-view attention-guided network (MAGN; deep learning Model)	0.785 ^2^		0.682–0.888 ^2^	
Liu et al. (2022) [15]	Differentiating spinal metastases from multiple myeloma	Radiomics (5EPV-16-model)	0.85 ^2^		-	
Liu et al. (2022) [23]	Differentiating osteolytic from osteoblastic spinal metastases	Radiomics	0.82 ^2^		0.71–0.93 ^2^	
Netherton et al. (2022) [24]	Automating treatment planning for diagnostic and simulation CT ^3^ scans	Deep learning (U-Net+) and random forest	0.82 ^2^		-	
Chianca et al. (2021) [25]	Spinal lesion differential diagnosis	Radiomics and machine learning (BaggedREPT ^4^)	0.90 ^1^		-	
Filograna et al. (2019) [26]	Identification of most significant predictors of metastasis	Deep learning—logistic regression model (T2-weighted images)	0.912 ^2^		0.829–0.994 ^2^	
Roth et al. (2015) [27]	Detection of sclerotic spine metastases	Deep learning—two-tiered cascade framework	0.834 ^2^		-	

**Table 5:** The AUC (external validation of each study’s best model) and corresponding 95% confidence interval of studies reporting AUC values for the established radiomics models for the external validation of the model is presented. AUCs range from 0.75 to 0.95. ^1^ = bagging combined with a REPTree.

Study	Output/Prediction	Best Performing Model	AUC	95% CI
Zijlstra et al. (2024) [11]	90-day survival	Machine learning algorithm (SORG-MLA)	0.81	0.77–0.86
	1-year survival		0.75	0.71–0.80
Duan et al. (2023) [17]	Differentiating spinal tuberculosis and spinal metastases	Multiscale vision transformers V2 (MVITV2)	0.95	-
Duan et al. (2023) [13]	Identification of origin for spinal metastases from MRI ^1^	Deep learning	0.76	-
Zhang et al. (2023) [14]	Differentiating spinal metastases from multiple myeloma	Radiomics nomogram	0.762	0.605–0.751
Chianca et al. (2021) [25]	Spinal lesion differential diagnosis	Radiomics and machine learning (BaggedREPT ^1^)	0.89	-

## Data Availability

All datasets used for this study are presented in the manuscript and its Supplementary Materials.

## Supplementary Materials

The following supporting information can be downloaded at https://www.mdpi.com/article/10.3390/jcm14165877/s1, Table S1: Search String; Table S2: Summary of included studies [10,11,12,13,14,15,16,17,18,19,20,21,22,23,24,25,26,27,32,33,36,40,41,42,43,44,45,46,47,48,49,50,51,52,53,54,55,56,57].
