# Supplementary material for: Applications and Performance of Artificial Intelligence in Spinal Metastasis Imaging: A Systematic Review
Source: J Clin Med. 2025 Aug 20;14(16):5877. doi: 10.3390/jcm14165877 (PMC12387603; doi:10.3390/jcm14165877)
Supplement: Supplementary file 1 [file jcm-14-05877-s001.zip › jcm-3721734-supplementary.pdf]

Supplementary Files

Supplementary Table S1 – Search queries across databases.

Database	No	Search Query	Results
EMBASE			
	#1	#2 AND **'article'**/it<br>**#1 ('artificial intelligence'**/exp OR **'artificial intelligence'** OR **'machine learning'**/exp OR **'machine learning'** OR **'deep learning'**/exp OR **'deep learning'** OR **'neural network'**/exp OR **'neural network'** OR **'artificial intelligence'**:ti,ab OR **'machine learning'**:ti,ab OR **'deep learning'**:ti,ab OR **'neural network'**:ti,ab OR **'radiomics'**:ti,ab OR **'predictive modeling'**:ti,ab OR **'computer assisted diagnosis'**:ti,ab) AND (**'spinal metastasis'**/exp OR **'spinal metastasis'** OR **'spine metastasis'**:ti,ab OR **'spinal metastasis'**:ti,ab OR **'metastatic spinal disease'**:ti,ab OR **'vertebral metastasis'**:ti,ab)	93
PubMed			
	#1	(("artificial intelligence"[MeSH Terms] OR "machine learning"[MeSH Terms] OR "deep learning"[MeSH Terms] OR "artificial intelligence"[Title/Abstract] OR "machine learning"[Title/Abstract] OR "deep learning"[Title/Abstract] OR "neural networks"[Title/Abstract] OR "radiomics"[Title/Abstract] OR "computer-assisted diagnosis"[Title/Abstract] OR "predictive modeling"[Title/Abstract]) AND ("spinal metastasis"[Title/Abstract] OR "metastatic spinal disease"[Title/Abstract] OR "spine metastasis"[Title/Abstract] OR "vertebral metastasis"[Title/Abstract] OR "metastatic spine disease"[Title/Abstract])) AND ((excludepreprints[Filter] OR medline[Filter]) AND (casereports[Filter] OR classicalarticle[Filter] OR clinicalstudy[Filter] OR clinicaltrial[Filter] OR multicenterstudy[Filter] OR observationalstudy[Filter] OR randomizedcontrolledtrial[Filter] OR technicalreport[Filter]) AND (humans[Filter]) AND (female[Filter] OR male[Filter]) AND (english[Filter]))	3
Scopus			
	#1	TITLE-ABS-KEY ("artificial intelligence" OR "machine learning" OR "deep learning" OR "neural network" OR "radiomics" OR "predictive modeling" OR "computer-assisted diagnosis" OR "AI-based diagnosis") AND TITLE-ABS-KEY ("spinal metastasis" OR "spinal metastases" OR "metastatic spinal disease" OR "spine metastasis" OR "vertebral metastasis") AND	120

		(LIMIT-TO (LANGUAGE , "English")) AND (LIMIT-TO (DOCTYPE , "ar"))	
Web of Science			
	#1	TS=("artificial intelligence" OR "machine learning" OR "deep learning" OR "neural network" OR "radiomics" OR "predictive modeling" OR "computer-assisted diagnosis" OR "AI-based diagnosis") AND TS=("spinal metastasis" OR "spinal metastases" OR "metastatic spinal disease" OR "spine metastasis" OR "vertebral metastasis") and Article (Document Types)	83
Cochrane			
	#1	("artificial intelligence" OR "machine learning" OR "deep learning" OR "neural networks" OR "radiomics" OR "predictive modeling" OR "computer-assisted diagnosis" OR "AI-based diagnosis") AND ("spinal metastasis" OR "spinal metastases" OR "metastatic spinal disease" OR "spine metastasis" OR "vertebral metastasis")	5

Supplementary Table S1. Search queries across five databases (PubMed, Scopus, Web of Science Advance, Cochrane, and Embase (Ovid)) are shown.

Supplementary Table S2 - Summary of the studies analyzed.

Sl No	Title	Year	Authors [ref]	Primary Tumor Type	Cohort Size	Number of lesions	Number of scans/ images	Type of Prediction Model (Deep Learning/ Machine Learning/ LLM)	Input/ Feature Variables	Output/ Prediction	Dice Similarity Coefficient (DSC)	Accuracy	Sensitivity	Specificity	AUC	Summary
1	ChatGPT's Performance in Spinal Metastasis Cases: Can We Discuss Our Complex Cases with ChatGPT?	2024	Heisinger, S. et al. (10)	Epitheloid sarcoma, pheochromo-cytoma, osseous leiomyo-sarcoma, anal squamous cell carcinoma, intracranial meningioma	5			Large Language Model (LLM)-ChatGPT-4	Clinical features of spinal metastases	Treatment recommendations (e.g., systemic therapy, pain management, supportive care, surgical intervention, palliative care)	NA	NA	Moderate to substantial agreement with experts (73%)	NA	NA	ChatGPT-4 showed alignment with expert recommendations in 73% of cases but provided generalized responses. Its performance improved in non-controversial areas, but lacked specificity in surgical and palliative care guidance.
2	Magnetic Resonance Imaging Frequency After Stereotactic Body Radiation Therapy for Spine Metastases	2024	Chen, H. et al.(40)	Breast 225, lung 209, kidney 191, prostate 164. Colon 57, Melanoma40, others 152, missing 1.	446		1039	Recursive Partitioning Analysis Model	Epidural disease, paraspinal disease, Spinal Instability Neoplastic Score, follow-up MRI intervals, progression rates	Risk-stratified MRI surveillance strategies based on spine progression risk and optimized imaging intervals	NA	NA	NA	NA	NA	A risk-stratification model categorized patients into low-, intermediate-, and high-risk groups for spine progression post-SBRT. Optimized MRI intervals were determined: low-risk every 6 months, intermediate-risk every 3 months, and high-risk every 1.3 months initially, based on progression rates.
3	Development of a natural language processing algorithm for the detection of spinal metastasis based on magnetic resonance imaging reports	2024	Mostafa, E. et al. (41)	NA	25,469		37,579	Neural Network (NLP)	MRI reports pre-processed using doc2vec algorithm, converted to numeric vectors	Likelihood of spinal tumor on MRI reports	NA	98.20%	100%	97.80%	NA	The study developed an NLP-based model to identify spinal tumors from MRI reports. The model was optimized for high sensitivity (100%) to minimize false negatives and showed a high accuracy of 98.2%. The model outperformed traditional untrained string searches, providing a significant improvement in specificity and PPV.
4	A New Deep Learning Algorithm for	2024	Motohashi, M. et al. (32)	Metastatic group Lung 65, kidney 31, colon 24,	447		475	Deep Learning (DL)-based AI Model	CT slices with annotated osteolytic	Automatic detection of osteolytic bone	NA	NA	78% (per slice), 75% (per lesion)	98% (per slice)	NA	This retrospective study developed a deep learning (DL)-based AI model to

	Detecting Spinal Metastases on Computed Tomography Images			Oesophagus 18, breast 17, Liver 13, hematologic 13, prostate 12, Pancreas 11, oral 10Skin 9, Miscellaneous (pharynx, stomach, bladder, uterus, thyroid, salivary glad, adrenal gland, thymus, testis, larnyx, small intestine, ovary, other) 32: Control group Metastatic group Lung 27, kidney 32, colon 33, Oesophagus 18, breast 7, Liver 2, MM 0, prostate 16, Pancreas 10, oral 3, skin 2 , Miscellaneous 42 (pharynx, stomach, bladder, uterus, thyroid, salivary gland, adrenal gland, thymus, testis, larnyx, small intestine, ovary, other)				lesions in the thoracolumbar spine	metastases in thoracolumbar CT scans						automatically detect osteolytic bone metastases in the thoracolumbar spine using CT scans. Trained on 263 positive and 172 negative scans from Tokyo Medical and Dental University Hospital, the model achieved a sensitivity of 0.78 and F1-score of 0.72 per slice. Observer studies showed the AI model's sensitivity was comparable to expert radiologists and orthopaedic surgeons, while improving detection performance among residents. Though further accuracy improvements are needed, the model shows potential for clinical application in detecting spinal metastases.
5	Improved localization and segmentation of spinal bone metastases in MRI with nnUNet radiomics	2024	Xu, Y. et al. (42)	NA	118		Deep Learning Model (nnUNet)	Manual physician-delineated tumor areas on MRI scans	Segmentation and identification of spinal bone metastases	Training set: 0.926, Test set: 0.824	NA	NA	NA	NA	The nnUNet radiomics model effectively localized and segmented spinal bone metastases on MRI scans. It achieved strong performance with Dice coefficients of 0.926 (training set) and 0.824 (test set). High correlation ($R^2 = 0.998$) was observed between model predictions and physician-delineated areas. Dice coefficients for lumbar and thoracic regions in the test set were 0.838 and 0.785, respectively. This study

															highlights the model's potential for automating tumor delineation in clinical practice.
6	AI-Assisted Detection and Localization of Spinal Metastatic Lesions	2024	Edelmers, E et al.(22)	Melanoma, lung, ovary, breast, prostate, kidney, GI, hematological, gastric	38		Deep Learning Model (U-Net)	CT images for vertebra and lesion segmentation	Detection and segmentation of spinal metastases	Vertebra segmentation: 0.87–0.96; Metastasis segmentation: 0.71 (lytic), 0.61 (sclerotic)	NA	NA	NA	NA	AI-based U-Net models achieved high DSC for vertebra segmentation (0.87–0.96). For metastases, lytic lesions showed a DSC of 0.71 and F-beta of 0.68, while sclerotic lesions were more challenging with DSC of 0.61 and F-beta of 0.57. The model also detected isolated metastatic lesions beyond the spine, showing potential for broader skeletal metastasis detection. The annotated CT dataset serves as a valuable resource for further advancements.
7	Uncertainty Quantification in Automated Detection of Vertebral Metastasis Using Ensemble Monte Carlo Dropout	2024	Ahn, SH et al. (16)	lung cancer (adenocarcinoma) (*n* = 21), breast cancer (*n* = 26), rectal cancer (*n* = 17), colon cancer (*n* = 22), stomach cancer (*n* = 8), tongue cancer (*n* = 3), malignant gastrointestinal stromal tumor (*n* = 2), pancreatic cancer (*n* = 2), appendiceal mucinous cystadenocarcinoma with pseudomyxoma peritonei (*n* = 1), thymic carcinoma (*n* = 1), adrenal cortical carcinoma (*n* = 1), renal cell carcinoma (*n* = 1),	127	12,425	Deep Learning Model (Ensemble Monte Carlo Dropout-EMCD)	Abdominal CT images; predictive probability interval, uncertainty maps	Detection of vertebral metastases with uncertainty quantification	NA	NA	NA	NA	0.93 (initial), 0.96 (with uncertainty-based data retention)<br>K-fold cross validation	The EMCD model integrates Monte Carlo dropout and deep ensembles for uncertainty quantification. It achieved an AUC of 0.93, improving to 0.96 with uncertainty-based data retention, and an expected calibration error of 0.09. This framework enhances the predictive reliability of vertebral metastasis detection and introduces uncertainty visualization to support clinicians in decision-making.

				Klatskin's tumor ($n = 1$), neuroendocrine carcinoma of tail of the pancreas ($n = 1$), ampulla of Vater cancer ($n = 1$), anaplastic hemangiopericytoma, malignancy ($n = 1$), esophagus cancer ($n = 1$), anal cancer ($n = 1$), nasopharyngeal cancer ($n = 1$), malignant melanoma ($n = 1$), hepatocellular carcinoma ($n = 1$), undifferentiated sarcoma ($n = 1$), and primary unknown ($n = 1$).											
8	An MRI-based radiomics nomogram for differentiating spinal metastases from multiple myeloma	2023	Zhang, S et al. (14)	Spinal Metastases lung (n = 94), breast (n = 18), liver (n = 13), kidney (n = 9), and esophageal (n =9), stomach (n=8), rectal (n=8), Colon (n=7), thyroid (n=5), Pancreatic (n=4), thymic (n=3), nasopharyngeal (n=2), endometrial (n=2), cervical (n=1), prostate. Multiple Myeloma =116	312		Radiomics Nomogram, Radiomics Signature Model, Clinical Model and one experienced radiologist	Demographics, MRI-derived radiomics features (heterogeneous appearance, shape, etc.)	Differentiation between spinal metastases and multiple myeloma	NA	Radiomics Nomogram: 68.3% (External)<br>Radiomics Signature Model: 67.3%(External)	Radiomics Nomogram: 78.8% (External)<br>Radiomics Signature Model: 71.2%(External)	Radiomics Nomogram: 67.1% (External)<br>Radiomics Signature Model: 63.3%(External)	Radiomics Nomogram: 0.856 (0.804-0.907) (training), 0.853(0.764–0.919) (validation), 0.762 0.605–0.751) (external)<br>Radiomics Signature Model :0.743 (0.668–0.861)	A radiomics nomogram combining radiomics-score, heterogeneous appearance, and shape was developed and validated. It demonstrated significantly better performance compared to clinical models and radiologists, with robust calibration and higher net benefit for differentiation of spinal metastases and multiple myeloma. Radiomics features were derived from T1WI and FS-T2WI images using LASSO regression. Sensitivity and specificity were superior to radiologists, particularly in external datasets.

														(validation), 0.754 (0.595–0.748) (external)	
											One Experienced radiologist: 73% (training), 66.2%(validation) ,63.4% (external)	One Experienced radiologist: 77% (training), 75%(validation) ,67.3% (external)	One Experienced radiologist: 65.2% (training), 47.6%(validation) ,59.2 (external)	Clinical Model: 0.715 (training), 0.692 (validation), 0.540 (external)	
														One Experienced radiologist: 0.711 (95%CI, 0.630–0.783) (training) , 0.613 (95%CI, 0.484–0.731)(validation) ,0.632 (95%CI, 0.531–0.726) (external)	
9	Contrast-enhanced magnetic resonance image segmentation based on improved U-Net and Inception-ResNet in the diagnosis of	2023	Wang, H. et al.(43)	Spinal Metastases 14 cases of lung cancer, 9 cases of breast cancer, 7 cases of prostate cancer, 6 cases of thyroid cancer, 5 cases of liver cancer, and 4 cases of renal cancer. vs.	81		Deep Learning (Improved U-Net, Inception-ResNet)	CE-MRI parameter maps, lesion segmentations	Differential diagnosis of spinal metastases and primary malignant spinal tumor	NA	Radiomics: 0.74, DL: 0.98	NA	NA	NA	This study used CE-MRI combined with radiomics and DL (improved U-Net and Inception-ResNet models) to classify spinal metastases vs. primary malignant spinal tumors. DL achieved superior diagnostic accuracy compared to radiomics.

	spinal metastases			Primary Malignant Spinal Tumor											
10	XGBoost-based multiparameters from dual-energy computed tomography for the differentiation of multiple myeloma of the spine from vertebral osteolytic metastases	2023	Shi, J. et al. (21)	Multiple Myeloma (MM) vs. Vertebral Osteolytic Metastases (VOM) lung adenocarcinoma (10 cases) and invasive breast cancer (13 cases)	51	137	XGBoost with multiparameter DECT (mpDECT)	Normalized effective atomic number, spectral Hounsfield unit slope, CT attenuation, virtual noncalcium (VNCa)	Classification of MM vs. VOM	NA	Training = 100%, Testing = 88%	Training = 100%, Testing = 80%	Training = 100%, Testing = 95%	Training: 1.00, Testing (external validation): 0.97	The study used XGBoost combined with mpDECT features to differentiate MM from VOM. The model outperformed univariate analysis and achieved excellent diagnostic performance with high specificity and AUC.
11	Deep learning-based magnetic resonance imaging of the spine in the diagnosis and physiological evaluation of spinal metastases	2023	Wang, D. et al. (44)	NA	941		Multilayer CNN + Softmax Classifier	Conventional MRI sequences (T1, T2)	Classification of spinal metastases into three categories (no metastasis, mild metastasis, apparent metastasis)	NA	96.45%	NA	NA	NA	A multilayer CNN model was trained on MRI sequences to classify spinal metastases into three categories. The highest accuracy achieved was 96.45%. The preprocessing steps included filtering, denoising, segmentation, and smoothing. The study highlights the potential of deep learning in early detection of spinal metastases.
12	MRI-based radiomics nomogram for differentiation of solitary metastasis and solitary primary tumor in the spine	2023	Li, S. et al. (19)	lung cancer (n = 29), renal cancer (n = 14), breast cancer (n = 7), rectal cancer (n = 5), prostate cancer (n = 2), stomach cancer (n = 2), thyroid cancer (n = 2), liver cancer (n = 1), esophageal cancer (n = 1), colon cancer (n = 1), pancreatic cancer (n = 1), cervical cancer (n = 1), bladder cancer (n = 1), ovarian cancer (n = 1),	135		Radiomics Nomogram (Machine Learning), Radiomics signature and Clinical model	Radiomics features + Clinical factors (Age, Signal, Boundaries)	Classification of Solitary Spinal Metastasis (SSM) vs. Solitary Primary Spinal Tumor (SPST)	NA	Radiomics Nomogram: Training: 93% Validation: 81%	Radiomics Nomogram: Training: 96% Validation: 82%	Radiomics Nomogram: Training: 90% Validation: 80%	Radiomics Nomogram: Training: 0.980 (0.959–0.995), External Validation: 0.924 (0.693–0.916)	A radiomics-based nomogram combining MRI radiomics features and clinical factors was developed to differentiate SSM from SPST. It achieved high diagnostic performance, with an AUC of 0.980 in the training set and 0.924 in the validation set, outperforming the clinical model.

submandibular gland cystadenocarcinoma (n = 1).				
	Radiomics signature: Training: 93% Validation: 81%	Radiomics signature: Training: 96% Validation: 88%	Radiomics signature:	Radiomics signature:
The SPST included plasmacytoma of bone (n = 12), giant cell tumor of bone (n = 11), chordoma (n = 10), lymphoma (n = 6), hemangioma (n = 5), osteoblastoma (n = 4), langerhans cell histiocytosis (n = 4), chondrosarcoma (n = 4), ewing sarcoma (n = 2), chondromyxoid fibroma (n = 2), liposarcoma (n = 2), aneurysmal bone cyst (n = 1), osteosarcoma (n = 1), epithelioid haemangioendothelioma (n = 1), fibrosarcoma (n = 1).			Training: 90% Validation: 75%	Training: 0.975 (0.950–0.993), Validation: 0.900 (0.702–0.914)
	Clinical model: Training: 69% Validation: 65%	Clinical model: Training: 67% Validation: 71%		
			Clinical model: Training: 71% Validation: 60%	Clinical model: Training: 0.807 (0.733–0.868), Validatio

															n: 0.679 (0.533–0.791)	
13	Accurate Differentiation of Spinal Tuberculosis and Spinal Metastases Using MR-Based Deep Learning Algorithms	2023	Duan, S. et al.(17)	37 cases of lung cancer, 8 cases of breast cancer, and 22 cases of other cancers (including liver, prostate and thyroid)	121	173	780	Deep Learning (MVITV2, EfficientNet-B3, ResNet101, ResNet34)	MRI-based deep learning features	STB vs. SM classification	NA	Internal Validation: MVITV2: 98.7%, EfficientNet-B3: 96.1%, ResNet101: 85.5%, ResNet34: 81.6%<br><br>External Validation: MVITV2: 91.9%, EfficientNet-B3: 85.9%, ResNet101: 80.8%, ResNet34: 78.8%	Internal Validation: MVITV2: 98.9%, EfficientNet-B3: 96.2%, ResNet101: 84.6%, ResNet34: 80.7%<br><br>External Validation: MVITV2: 91.5%, EfficientNet-B3: 91.1%, ResNet101: 84.4%, ResNet34: 82.2%	NA	Internal Validation: MVITV2: 0.98, EfficientNet-B3: 0.99, ResNet101: 0.90, ResNet34: 0.85 External Validation: MVITV2: 0.95, EfficientNet-B3: 0.91, ResNet101: 0.87, ResNet34: 0.86	The study evaluates deep learning models for differentiating spinal tuberculosis (STB) from spinal metastases (SM) using T2-weighted sagittal MRI scans. MVITV2 outperformed other models, achieving an AUC of 0.98 (internal) and 0.95 (external), with diagnostic accuracy comparable to an experienced spine surgeon. The findings highlight DL as a potential assistive tool in clinical decision-making.
14	Differentiation of predominantly osteolytic from osteoblastic spinal metastases based on standard magnetic resonance imaging sequences: a comparison of radiomics model versus semantic features logistic regression model findings	2022	Liu, K. et al. (23)	Spinal Metastases (Osteolytic vs. Osteoblastic Differentiation) Lung 24, renal 11, breast 8, prostate 7, Cancer of unknown origin 7, Colorectal 5, hepato cellular 5, cholangio carcinoma 2, Rhabdomyosarcoma 1, Leiomyosarcoma 1. thyroid	78	78		Machine Learning (Logistic Regression, SVM Feature Selection)	107 radiomics features (6 selected via SVM) + 5 semantic MRI features	Prediction of osteolytic vs. osteoblastic spinal metastases	N/A	Radiomics Model: 78.2% Semantic Features Model: 75.6%	Radiomics Model: 73.1% Semantic Model: 80.8%	Radiomics Model: 80.8% Semantic Model: 73.1%	Radiomics Model: 0.82 (0.71–0.93) Semantic Model: 0.79 (0.68–0.91)<br><br>10-fold cross validation	The standard MRI-based radiomics model outperformed the semantic features logistic regression model in differentiating predominantly osteolytic and osteoblastic spinal metastases. The radiomics model showed an AUC of 0.82, accuracy of 78.2%, sensitivity of 73.1%, and specificity of 80.8%, whereas the semantic feature model had an AUC of 0.79, accuracy of 75.6%, sensitivity of 80.8%, and specificity of 73.1%. CT was used as a reference for classification accuracy.

15	Differentiation between spinal multiple myeloma and metastases originated from lung using multi-view attention-guided network	2022	Chen, K. et al. (22)	Spinal Multiple Myeloma 81vs. Metastases 188(Lung Cancer Originated)	217	269	Deep Learning (Multi-view Attention-Guided Network - MAGN), compared with Radiomics (Random Forest-based Multi-view Model) and Radiologist Assessment	MAGN: Multi-view deep learning features + attention-guided classification Radiomics: 768 features (30 selected using random forest) from sagittal, axial, and coronal MRI views	Differentiation between spinal MM and metastases	N/A	MAGN: 81% Radiomics: 71% Radiologist (10-year experience) : 69%	NA	NA	Sagittal – 0.7546±0.1011 √ 0.8061 0.7492±0.0671 Axial – 0.7020±0.0268 0.7739 0.7196±0.0441 Coronal – 0.6642±0.0808 0.7322±0.1112 Multi-view – 0.7661±0.0841 0.7847±0.1030 Radiomics model Sagittal – 0.6438 ± 0.0445 Axial - 0.6417 ± 0.0651 Coronal - 0.6587 ± 0.0514 Multi-View - 0.7616 ± 0.0386 Radiologist 1=0.6147 Radiologist 2=0.7085	MAGN outperformed radiomics-based and radiologist assessments in differentiating spinal MM from metastases, achieving the highest diagnostic accuracy (81%) and AUC (0.78). The radiomics model, using 30 selected features from multi-view MRI analysis, performed better than single-view classifiers (AUC 0.76). Radiologist performance was slightly lower (AUC 0.71). This highlights the advantage of deep learning for feature extraction and classification in spinal tumor differentiation. 5-fold cross validation
16	Effectiveness of temporal subtraction	2022	Hoshiai, S. et al.(45)	Lung, Breast, colon, oesophagus, skin, ovary, kidney,	40		Deep Learning-temporal subtraction	CT images with and without	Detect vertebral bone metastasis	NA	NA	NA	FOM Radiologists	overall FOM with and	TS CT was effective in detecting bone metastasis by both board-certified

	computed tomography images using deep learning in detecting vertebral bone metastases			uterus, liver, bladder, stomach, pancreas, pharynx, testis, prostate, retro-peritoneum, anus, perineum			computed tomography (TS CT)	temporal subtraction					Without TSCT 0.848 (0.796–0.899) With TSCT 0.876 (0.829–0.923) FOM Residents Without TSCT 0.752 (0.680–0.824) With TSCT 0.799 (0.744–0.855)	without TS CT was 0.876 (95% confidence interval [CI]: 0.829–0.923) and 0.848 (95 %CI: 0.796–0.899), respectively, in the experienced radiologist group and 0.799 (95 %CI: 0.744–0.855) and 0.752 (95 %CI: 0.680–0.824), respectively, in the resident group	radiologists and radiology residents.
17	Vertebral MRI-based radiomics model to differentiate multiple myeloma from metastases: influence of features number on logistic regression	2022	Liu, J. et al.(15)	Vertebral Multiple Myeloma 103 VS Metastasis 138 (lung 79,prostate 15, breast 14, renal 9, liver 7, thyroid 6,esophageal 5,pancreas 2, melanoma 1.	241		Machine Learning (Logistic regression, volumes of interest)	MRI (T1WI and fat-suppression T2WI) images	To differentiate MM from metastasis	NA	Validation: 20EPV-4-Model 66.9%, 15EPV-6-Model 74.7%, 10EPV-8-Model 78.6%, 5EPV-16-Mode	Validation: 20EPV-4-Model 72.1%, 15EPV-6-Model 75.4%, 10EPV-8-Model 82%, 5EPV-16-Mode 81.7%, 2EPV-CFS-Model 79.2%.	Validation: 20EPV-4-Model 60%, 15EPV-6-Model 73.7%, 10EPV-8-Model 74%, 5EPV-16-Mode	Testing	The radiomics model constructed with an appropriate small number of the most frequent features could well distinguish metastases from MM based on conventional vertebral MRI. Based on our results, we recommend following the 10 EPV as the rule of

model performance		80.1%, 2EPV-CFS-Model 73.5%.		78.1%, 2EPV-CFS-Model 65.8%.		thumb for feature selection.
		Training:	Training: 20EPV-4-Model 84.1%, 15EPV-6-Model 76.4%, 10EPV-8-Model 84.5%, 5EPV-16-Mode 85.5%, 2EPV-CFS-Model 86.7%.	Training: 20EPV-4-Model 48.9%, 15EPV-6-Model 78.4%, 10EPV-8-Model 77.7%, 5EPV-16-Mode 81%, 2EPV-CFS-Model 85.1%.	20EPV-4-Model 0.73, 15EPV-6-Model 0.84, 10EPV-8-Model 0.86, 5EPV-16-Mode 0.89, 2EPV-CFS-Model 0.94.	
		20EPV-4-Model 69%, 15EPV-6-Model 77.2%, 10EPV-8-Model 81.5%, 5EPV-16-Mode 83.5%, 2EPV-CFS-Model 86%.			Validation	
					20EPV-4-Model -0.71, 15EPV-6-Model 0.81, 10EPV-8-Model 0.84, 5EPV-16-Model 0.85, 2EPV-CFS-	

														Model 0.78.	
18	Radiomics in Breast Cancer: In-Depth Machine Analysis of MR Images of Metastatic Spine Lesion	2022	Steinhauer, V. et al. (46)	Breast Cancer=3	3	12	Machine Learning	MRI images	To detect metastatic spain lesions from breast cancer	NA	NA	NA	NA	NA	The study has shown a high effectiveness of machine image analysis algorithms, high correlation of the obtained results with the radiologist's report and clinical and laboratory data in 9 cases out of 12. The Pearson correlation coefficient between the classical marker and matrix filter curve was 0.8.
19	Automated detection and segmentation of sclerotic spinal lesions on body CTs using a deep convolutional neural network	2022	Chang, C.Y. et al. (47)	NA		600	Deep learning - convolutional neural network	background, normal bone, and sclerotic lesion(s).	Automated detection and segmentation of sclerotic spinal lesions	0.83 for lesion, 0.96 for non-pathologic bone, and 0.99 for background	NA	Global sensitivity was 95%, local sensitivity was 92%	local specificity was 87%		A deep convolutional neural network has the potential to assist in detecting sclerotic spinal metastases.
20	A Texture Analysis Approach for Spine Metastasis Classification in T1 and T2 MRI	2018	Larhmam MA. et al. (48)	NA	142	NA	Machine learning- data normalization, cross validation and feature selection	T1 and T2 MRI Spine sagittal sections	Classifying spine metastasis	NA	T1 82.88% and SD 5.57; T2 88.86% and SD 3.35; T1+T2 90.08% and SD 5.65	NA	NA	NA	This study proposed a learning-based method to classify pathological vertebrae in spine metastases using MRI images. Texture analysis techniques were applied to co-registered T1 and T2 MRI sequences, commonly used for vertebral metastasis follow-up. The model analyzed 153 vertebrae slice by slice, selecting 67 relevant features from an initial set of 142. The method demonstrated high classification accuracy, aiding in rapid diagnosis and treatment monitoring of spinal metastases.
21	Deep Learning on MRI Images for Diagnosis of Lung Cancer	2021	Fan, X. et al. (49)	Lung cancer=87	87		Deep Learning- AdaBoost Algorithm (Adaptive Boosting), Chan-	CT images	To detect spinal metastasis from lung cancer	Dice index and Jaccard coefficient of the OTSU algorithm were 0.6125 and 0.5541,	The accuracy of level I image classificati	2.30%	NA	NA	In summary, the AdaBoost algorithm was adopted for image preliminary classification, and CV algorithm for

	Spinal Bone Metastasis						Vese (CV) Algorithm and OTSU			respectively.; region growing algorithm were 0.7293 and 0.6598, and Jaccard coefficient of the CV algorithm were 0.8591 and 0.8002, respectively.	on was 89.4%, accuracy of level II image classificati on was 87.9%, that of level III image classificati on was 92.1%,				image segmentation was ideal for the diagnosis of lung cancer spinal bone metastasis and it was worthy of clinical promotion.
22	Texture Analysis of 18F-FDG PET/CT for Differential Diagnosis Spinal Metastases	2021	Fan, X. et al.(50)	NA	89	NA	**Machine Learning** techniques with three classification algorithms: **Logistic Regression, Support Vector Machine (SVM), Decision Tree**	PET/CT images	To evaluate the value of texture analysis for the differential diagnosis of spinal metastases	NA	The accuracy of classificati on in the test set was 87.5, 83.34, and 75%, respectivel y in logistic regression, decision tree, and support vector machine. Manual diagnosis: 84.27%	SUL peak 90.9 %<br>Correlation 84.8%<br>Intensity 75.8%<br>SUV variance 78.8%<br>Maximum SUV 69.7%	SUL peak 65.6%<br>Correlatio n 75%<br>Intensity 81.2%<br>SUV variance 75%<br>Maximum SUV 84.4%	SUL peak 0.831 (0.728, 0.935)<br>Correlati on 0.827 (0.719, 0.935)<br>Intensity 0.82 (0.715, 0.925)<br>SUV variance 0.817 (0.709, 0.926)<br>Maximu m SUV 0.806 (0.696, 0.915)	Partial texture features showed higher diagnostic value for spinal metastases than SUVmax. The machine learning part of the model combined with the texture parameters was more accurate than manual diagnosis. Therefore, texture analysis may be useful to assist in the diagnosis of spinal metastases.
23	Identification of the most significant magnetic resonance imaging (MRI) radiomic features in oncological patients with vertebral bone marrow metastatic	2019	Filograna, L. et al. (26)	3 lung cancer; 1 prostatic cancer; 1 esophageal cancer; 1 nasopharyngeal cancer; 1 hepatocarcinoma; 1 breast cancer	8	29	Deep Learning-Logistic Regression Model	T1/T2 MRI images	Detecting most significant magnetic resonance imaging (MRI) radiomic features in oncological patients with vertebral bone marrow metastatic disease	NA	NA	NA	NA	Internal cross validatio n: 0.8141 (95% CI 0.6854–0.9427) in T1 images and 0.9116 (95% CI	The results suggest that MRI-based radiomic analysis on oncological patients with bone marrow metastatic disease is able to differentiate between metastatic and non-metastatic vertebral bodies. The most significant predictors of metastasis were found to be based on T2 sequence and were one

	disease: a feasibility study														0.8294–0.9937) in T2 images.	morphological and one textural feature.
24	Deep convolutional neural network-based segmentation and classification of difficult to define metastatic spinal lesions in 3D CT data	2018	Chmelik, J.et al.(51)	NA				Deep Learning-convolutional neural network (CNN)	CT images	Detecting spinal metastasis	NA	NA	Lytic lesions 0.71, sclerotic 0.74	Lytic lesions 0.88, sclerotic 0.92	Lytic lesions 0.80, sclerotic 0.78	Our proposed method has been tested on our own dataset annotated by two mutually independent radiologists and has been compared to other published methods. This work is part of the ongoing complex project dealing with spine analysis and spine lesion longitudinal studies.
25	A multi-resolution approach for spinal metastasis detection using deep Siamese neural networks	2017	Wang, J. et al. (33)	15 lung, 5 thyroid, two liver, one breast, one prostate, one esophagus, one urinary tract.	26			Deep Learning-Siamese neural networks	multiple sets of MRI images	Detecting spinal metastasis	NA	NA	72.7% (without aggregation)<br>89.1% (with aggregation)	NA	NA	At a true positive (TP) rate of 90%, the use of the aggregation reduces the FPs from 0.375 FPs per case to 0.207 FPs per case, a nearly 44.8% reduction. The results indicate that the proposed Siamese neural network method, combined with the aggregation strategy, provide a viable strategy for the automated detection of spinal metastasis in MRI images.
26	Mixed spine metastasis detection through positron emission tomography/computed tomography synthesis and multi classifier	2017	Yao, J. et al. (52)	NA	44	456	44	Computer aided detection	CT and PET scans	Spinal metastasis detection using imaging	NA	NA	81%, 81%, 76% for lytic, sclerotic, mixed respectively	NA	NA	The system was tested on 44 cases with 225 sclerotic, 139 lytic, and 92 mixed lesions. The results showed that sensitivity (false positive per patient) was 0.81 (2.1), 0.81 (1.3), and 0.76 (2.1) for sclerotic, lytic, and mixed lesions, respectively. It also demonstrates that using PET/CT data significantly improves the computer aided detection performance over using CT alone.
27	Thoracic temporal subtraction	2017	Iwano, S. et al.(53)	Lung cancer=30	30	46	30	Computer aided detection (CAD)	CT images	To detect vertebral metastases on the follow-up CT images of	NA	NA	Reader A: CAD (–) 0.717, CAD (+) 0.826,	Reader A: CAD (–) 0.981,	Reader A:	temporal 3D-CT subtraction CAD software easily detected vertebral

	three-dimensional computed tomography (3D-CT): Screening for vertebral metastases of primary lung cancers									lung cancer patients regardless of the osteolytic or osteoblastic nature of the lesions.			Reader B: CAD (-)0.783, CAD (+) 0.848.	CAD (+) 0.974, Reader B: CAD (-)0.995, CAD (+) 0.993.	CAD (–) 0.849, CAD (+) 0.902, Reader B: CAD (-)0.889, CAD (+) 0.910.	metastases on the follow-up CT images of lung cancer patients regardless of the osteolytic or osteoblastic nature of the lesions. This software could prevent radiologists from overlooking asymptomatic vertebral metastases during routine interpretation of thoracic CT images for follow-up of lung cancer.
28	Detection of sclerotic spine metastases via random aggregation of deep convolutional neural network classifications	2015	Roth, H.R. et al.(27)	NA	59	532		Deep Learning-two-tiered cascade framework	CT images	To detect sclerotic vertebral metastasis using CT images	NA	NA	First Tier model: 92%	NA	0.834 k-fold cross validation	this work demonstrates that deep CNNs can be generalized to tasks in medical image analysis, such as effective FP reduction in computer-aided detection (CADe)) systems.
29	Lytic metastases in thoracolumbar spine: Computer-aided detection at CT - Preliminary study	2007	O'Connor, S.D. et al. (54)	Melanoma (n = 19), renal cell carcinoma (n = 10), prostate cancer (n = 4), lung cancer (n = 4), lymphoma (n = 2), breast cancer (n = 2), pheochromocytoma (n = 2), or other disorders (n = 7)	50			Computer aided detection (CAD)	CT images	To identify probable lytic metastases in the thoracolumbar spine	NA	NA	Training group sensitivity 0.83 (95% confidence interval: 0.51, 0.97). Test set sensitivity 0.94 (95% confidence interval: 0.68, 1.00)	NA	NA	This CAD system successfully identified probable lytic metastases in the thoracolumbar spine and generalized well to an independent testing set.
30	An Automated Treatment Planning Framework for Spinal Radiation Therapy and Vertebral-Level Second Check	2022	Netherton, T.J. et al. (24)	NA			336	Deep Learning (U-Net++ for segmentation) and Random Forest (classification)	CT-based auto-contours and anatomical features	Automated vertebral labeling, contouring, and treatment planning	Cervical: 85.0% Thoracic: 90.3% Lumbar: 93.7%	0.82 (all vertebrae) 0.83 (target regions only)	0.82 (all vertebrae), 1.00 (target regions only)	0.82 (all vertebrae), 0.80 (target regions only)	0.82 (random forest classifier for mislabeling prediction) k-fold cross validation	This study developed a fully automated tool for vertebral labeling, segmentation, and treatment planning using U-Net++ and a random forest classifier. The tool effectively labeled and segmented vertebrae from C1-L5 across various CT scan types, with high DSC scores (85-94%). The random forest model predicted vertebral mislabelling with AUC = 0.82. Radiation oncologists rated 98% of

																simulation CT-based plans and 92% of diagnostic CT-based plans as clinically acceptable or requiring minor edits. The end-to-end treatment planning process took less than 8 minutes, demonstrating its potential for fast and accurate clinical implementation.
31	CT-based finite element simulating spatial bone damage accumulation predicts metastatic human vertebrae strength and stiffness	2024	Soltani, Z. et al. (55)	Breast (4), Lung (3), Prostate (2), Kidney (1)	9	10	CT data (number unspecified)	Finite Element (FE) Framework (damage-based)	X-Ray CT-derived specimen-specific and globally calibrated finite element models	Prediction of vertebral strength, stiffness, and damage evolution patterns	NA	$R^2 = 0.99$ (specimen-specific), $R^2 = 0.83$ (global)	NA	NA	NA	This study developed a finite element (FE) framework to predict vertebral strength and stiffness from CT data in patients with spinal metastases. The model accurately predicted fracture risk using both global and specimen-specific material calibration, with near-perfect results for the latter ($R^2 = 0.99$). The simulations revealed that fractures primarily originate in low bone density regions, offering a potential tool for early, image-based diagnosis of impending pathologic vertebral fractures.
32	Bone density measurement in patients with spinal metastatic tumors using chest quantitative CT deep learning model	2024	Wang, Z. et al.(12)	NA	749		749	Deep Learning (3DResUNet)	QCT scans with automated vertebral segmentation and ROI extraction	Vertebral volumetric bone mineral density (vBMD) prediction and osteoporosis diagnosis	NA	NA	NA	NA	Training: 0.977 (95 % CI: 0.970–0.984) ; Test (external validation): 0.966 (95 % CI: 0.944–0.988)	This study developed a deep learning model using the 3DResUNet architecture to predict vertebral vBMD from QCT scans in patients with spinal metastatic tumors. The model demonstrated high correlation (Spearman coefficients of 0.923 and 0.918 for training and test sets, respectively) with QCT-measured vBMD and excellent performance in diagnosing

															osteoporosis, achieving AUCs of 0.977 (training set) and 0.966 (test set). It offers accurate, automated vBMD predictions, facilitating osteoporosis screening in clinical practice.
33	Temporal validation of the SORG 90-Day and 1-Year machine learning algorithms for survival of patients with spinal metastatic disease	2024	Zijlstra, H. et al.(11)	NA	464		Machine Learning Algorithm (SORG-MLA)	18 variables, including primary tumor type, ECOG performance status, and nine preoperative lab values	90-day and 1-year postoperative survival predictions	NA	NA	NA	NA	90-day: 0.81 (CI: 0.77–0.86), 1-year: 0.75 (CI: 0.71–0.80)	The study validated the SORG-MLA for predicting 90-day and 1-year postoperative survival in patients with spinal metastatic disease, using a contemporary cohort from 2017–2021. Despite differences between development and validation cohorts, the algorithm performed well on calibration, discrimination (AUC of 0.81 for 90-day and 0.75 for 1-year survival), and decision curve analysis. The study suggests potential for improving the model by updating with recent data and stratifying by primary tumor.
34	Identification of Origin for Spinal Metastases from MR Images: comparison Between Radiomics and Deep Learning Methods	2023	Duan, S et al.(13)	breast cancer (30), melanoma (11), prostatic cancer (9), rectum cancer (24), liver cancer (10), kidney cancer (11), and thyroid cancer (10), lung cancer	173	Not specified	Deep Learning (DL) and Radiomics (RAD) Models (Support vector machine-SVM and XGboost)	Contrast-enhanced T1 MR images from spinal metastases	Classification of metastasis origin (lung cancer vs. other cancer types)	NA	Training: DL: 0.93, RAD (SVM): 0.84, RAD (XGboost) : 0.80<br><br>Validation: 0.74, RAD (SVM): 0.72, RAD (XGboost) : 0.66<br><br>Test: DL: 0.72, RAD	NA	NA	Training: DL: 0.94, RAD (SVM): 0.93, RAD (XGboost) :0.90 Validation: 0.76, RAD (SVM): 0.75, RAD (XGboost) :0.73 Test: DL: 0.76,	The study developed DL and RAD models to classify the origin of spinal metastases from lung or other cancers based on CET1-MR images. The DL model consistently outperformed RAD and expert radiologists, achieving high accuracy and AUC, though external validation showed slightly reduced performance.

											(SVM): 0.69			RAD (SVM): 0.72	
35	Artificial intelligence-aided lytic spinal bone metastasis classification on CT scans	2023	Koike, Y. et al.(18)	NA	79	2125	Deep Learning-based CAD System	Whole CT images, vertebra bounding boxes, lytic lesion presence/absence	Detection and classification of lytic spinal bone metastasis	IoU: 0.923 ± 0.052 (0.684-1.000)	87.20%	74.10%	94.80%	Test (external validation) dataset= 0.941 Each fold in cross validation = > 0.95	artificial intelligence-aided CAD system using two DL models could rapidly identify vertebra bone from whole CT images and detect lytic spinal bone metastasis, although further evaluation of diagnostic accuracy is required with a larger sample size.
36	Prediction of Primary Tumor Sites in Spinal Metastases Using a ResNet-50 Convolutional Neural Network Based on MRI	2023	Liu, K. et al.(20)	lung cancer (*n* = 142), kidney cancer (*n* = 50), mammary cancer (*n* = 41), thyroid cancer (*n* = 34), and prostate cancer (*n* = 28	295		ResNet-50 CNN (Deep Learning)-5-class classifiers, 4-class classifiers, and 3-class classifiers	Conventional MRI sequences (T1, T2, Fat-suppressed T2)	Classification of primary tumor site from spinal metastases	NA	5-class: 52.97%; 4-class: 58.46%; 3-class: 67.16%	5-class: 48.56%; 4-class: 57.13%; 3-class: 66.91%	5-class: 61.81%; 4-class: 80.77%; 3-class: 83.97%	5-class: 0.77 (0.76~0.77); 4-class: 0.81(0.80~0.82); 3-class: 0.85 (0.84~0.86) k-fold cross (AUC: 0.77 average) validation and external validation	ResNet-50 CNN model was trained on MRI sequences to predict primary tumor sites in spinal metastases. Performance varied across classification tasks, with the highest AUC of 0.85 for the 3-class model.
37	Radiomic Machine Learning Classifiers in Spine Bone Tumors: A Multi-Software, Multi-Scanner Study	2021	Chianca, V. et al.(25)	Spine tumours	146	146	Radiomics and machine learning. 2 label classification: Deep artificial neural network (D-ANN) and BaggedREPT (Bagging	MRI based images	ML diagnostic performance in spinal lesion differential diagnosis	NA	ML, 94% accuracy in the internal test cohort, and 86% in the external one. For the 3-label	Multi-scanner external test cohort, D-ANN and baggedREPT correctly identified 71% (25/35) and 86% (30/35) of lesions in the 2-label classification, respectively. On the other hand, IOLB	Multi-scanner external test cohort, D-ANN and baggedREPT correctly identified	**2 label classification:** Internal test:	MRI radiomics combined with ML may be useful in spinal lesion assessment. More robust pre-processing led to better consistency despite scanner and protocol heterogeneity.

						combined with REPTree)<br><br>3 label classification: Iteratively optimised Logit boost (IOLB) and BoostJ48				classificati on, PyRadiomi cs data, 80% and 69% accuracy in the internal and external test sets, respectivel y.	achieved 60% (21/35) and boostJ48 69% (24/35) accuracy in the 3-label classification.	71% and 81% of lesions in the 2-label classificati on, respectivel y. On the other hand, IOLB achieved 80% and 85% accuracy in the 3-label classificati on.	D-ANN:0.9 0, BaggedR EPT :0.9 0<br><br>External test: D-ANN:0.7 0, BaggedR EPT :0.8 9<br>**3 label classific ation:**<br>Internal test: IOLB:0.7 3, BoostJ48 :0.89<br><br>External test: IOLB:0.8 3, BoostJ48 :0.83	
38	Differentiation of spinal metastases originated from lung and other cancers using radiomics and deep learning	2019	Lang, N. et al. (56)	30 lungs; 31 non-lung cancers, breast, thyroid,, prostate, liver, renal	61	Deep Learning- conventional convolutional neural network (CNN) AND convolutional long short-term memory (CLSTM) network.	DCE parametric maps and 12 sets of DCE images	Differentiation of spinal metastases originated from lung and other cancers	NA	CNN 0.71 ± 0.043, CLSTM 0.81 ± 0.034, Radiomics 0.71, CHAID classificati	NA	NA	NA	DCE-MRI machine-learning analysis methods have potential to predict lung cancer metastases in the spine, which may be used to guide subsequent workup for confirmed diagnosis.

	based on DCE-MRI										on 0.79, logistic regression 0.74				
39	Evaluation of a Multiview architecture for automatic vertebral labeling of palliative radiotherapy simulation CT images	2020	Netherton, T.J. et al. (57)	NA	330		Deep Learning- X net, X net ensemble, Btrfly Net	CT images	To automatically label vertebral levels (S2-C1) in palliative radiotherapy simulation CT scans.	NA	X-Net: **92.4%** X-Net Ensemble: **94.2%** Btrfly Net: **90.5%** X-Net Ensemble in the normative group: **96.9%**	67% (for the **method to detect irregular intervertebral spacing)**	97% (for the **method to detect irregular interverteb ral spacing)**	NA	X-Net, a unique convolutional neural network, to automatically label vertebral levels from S2 to C1 on palliative radiotherapy CT images showed that an ensemble of X-Net models had high vertebral body identification rate (94.2%) and small localization errors (2.2 ± 1.8 mm). In addition to this, transfer learning approach achieved state-of-the-art results on a well-known benchmark dataset with high identification rate (91.3%) and low localization error (3.3 mm ± 2.7 mm)..

Supplementary Table S2. A summary of the studies focusing on diagnosis and image analysis in spinal metastases is presented.
